# The Effects of Metabolic and Bariatric Surgery on the Anterior Segment Parameters of the Eye

**DOI:** 10.1007/s11695-025-07773-0

**Published:** 2025-03-14

**Authors:** Muslum Toptan, Hasan Elkan, Hamza Erdogdu, Omer Goc, Omer Faruk Yilmaz

**Affiliations:** 1 Department of Ophthalmology, Harran University, Şanlıurfa, Turkey; 2 Department of General Surgery, Harran University, Şanlıurfa, Turkey; 3 Department of Biostatistics, Harran University, Şanlıurfa, Turkey; 4 Vocational School of Health Services, Biomedical Device Technologies, Harran University, Şanlıurfa, Turkey; 5 Department of Ophthalmology, Medeniyet University Göztepe Training and Research Hospital, Istanbul, Turkey

**Keywords:** Anterior segment parameters, Anterior chamber depth, Anterior chamber volume, Anterior chamber angle, Body mass index, Metabolic and bariatric surgery

## Abstract

**Purpose:**

This study aimed to investigate the effects of metabolic and bariatric surgery (MBS) on the corneal and anterior segment parameters of the eye.

**Materials and Methods:**

In this study, the anterior chamber depth, volume, angle and central corneal thickness of obese patients who underwent laparoscopic sleeve gastrectomy surgery were prospectively examined using Pentacam topography before and six months after surgery.

**Results:**

A total of 112 eyes of 56 individuals, including 26 males with a mean age of 38.88 ± 7.00 years and 30 females with a mean age of 40.94 ± 7.76 years, were evaluated (*p* = 0.310). Six months following MBS, the average body mass index (BMI) in men decreased from 45.5 kg/m^2^ to 34 kg/m^2^ (*p* < 0.001), and the BMI decreased from 47 kg/m^2^ to 33 kg/m^2^ (*p* < 0.001) in women. Central corneal thickness and intraocular pressure decreased significantly in both men and women. The anterior chamber depth, volume and angle ​​significantly increased six months after surgery (*p* < 0.001 for all parameters). A statistically significant negative correlation was observed between postoperative BMI and anterior chamber depth, volume and angle in both sexes (*p* < 0.001 for all).

**Conclusion:**

This study revealed that anterior segment values, intraocular pressure, and corneal thickness may change after MBS. Caution should be exercised when evaluating for cataracts and refractive surgery after MBS.

**Graphical Abstract:**

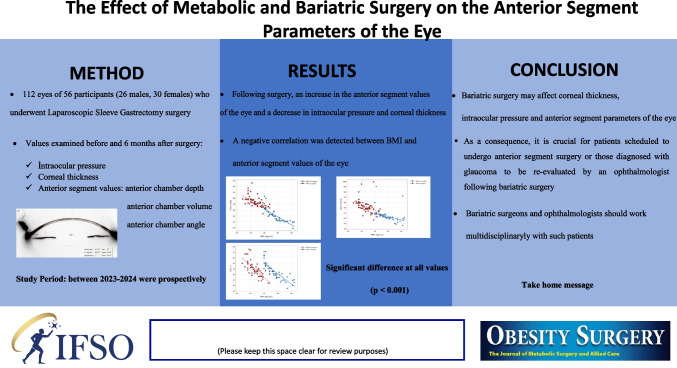

## Introduction

Obesity is a widespread global public health issue. It frequently results in retinal pathologies due to associated conditions such as type 2 diabetes mellitus, cardiovascular disease, dyslipidaemia, and hypertension [1–3]. Various studies have shown differences in anterior segment parameters and intraocular pressure between obese individuals and healthy individuals [4]. The assessment of anterior segment parameters, including anterior chamber depth, anterior chamber volume, anterior chamber angle and central corneal thickness, is highly important in ophthalmic evaluations. These metrics offer invaluable insights for tasks such as assessing the risk of glaucoma, calculating intraocular lens power, monitoring keratoconus progression, and investigating refractive disorders [5].

Laparoscopic sleeve gastrectomy (LSG) has become a commonly employed surgical approach in light of the escalating obesity epidemic [3]. Studies are needed to investigate the extent to which weight loss resulting from surgical intervention in obese patients affects the anterior segment parameters associated with a reduction in fat around the orbit. In this study, changes in the corneal and anterior segment parameters of obese patients who underwent LSG were investigated using Pentacam topography.

## Materials and Methods

A total of 112 eyes from 56 obese patients (26 men and 30 women) aged 18–60 years with a body mass index (BMI) ≥ 40 kg/m^2^ who underwent LSG surgery at Harran University General Surgery Clinic between 2023 and 2024 were examined prospectively. The study was conducted in accordance with the principles of the Declaration of Helsinki and was approved by the local ethics committee (231,738). Written informed consent was obtained from all the subjects. Comprehensive physical examinations, anthropometric calculations, and blood pressure measurements were performed in the obesity unit. BMI was calculated via anthropometric data (weight, height) and the formula weight (kg)/height^2^ [2]. Blood glucose (g/dL), glycosylated haemoglobin (HbA1c, %), triglycerides (mg/dL), total cholesterol (mg/dL), low-density lipoprotein (LDL, mg/dL), and high-density lipoprotein (HDL, mg/dL) levels were measured in fasting blood samples using standard laboratory test evaluations before and six months after surgery.

A comprehensive ophthalmic examination, including slit lamp biomicroscopy, tonometry, retinoscopy, and topographic evaluation, was performed. Best corrected visual acuity measurements were recorded in Snellen equivalents for both the right eye and left eye during visits to the ophthalmology clinic, both before and six months following MBS. Intraocular pressure was measured by the same glaucoma specialist (MT) using a Goldmann applanation tonometer (Haag-Streit, Koeniz, Switzerland), taking into account the average of two measurements at specific time intervals (08:00–10:30). Central corneal thickness, anterior chamber depth, volume and angle were assessed using Scheimpflug Pentacam topography (Pentacam, Oculus, Inc., Lynnwood, WA, USA). These measurements were performed by a single experienced technician under scotopic conditions, with the pupils undilated. The average intraocular pressure, central corneal thickness, anterior chamber depth, and volume and angle values ​​for the right and left eyes were obtained.

LSG for MBS involves creating a vertical gastric tube over a 39 French orogastric tube, starting 2–4 cm from the pylorus and ending approximately 2 cm lateral to the angle of His using a stapler. The staple line of all patients was sutured with omentopexy. Blood tests were performed to monitor vitamin deficiency during routine follow-up in all patients before and after surgery. If deficiency was detected, B1 (thiamine), B6 ​​(pyridoxine), the B12 vitamin complex, other vitamins and folic acid supplements were administered orally.

## Exclusion Criteria

Patients with obstructive sleep apnoea, type 2 diabetes mellitus, hypertension, hyperlipidaemia, systemic steroid use that may affect intraocular pressure, cataracts, glaucoma, ocular hypertension, uveitis, any type of retinopathy, a history of ophthalmic surgery, corneal pathology, and refractive errors between + 2 D and −2 D based on the spherical/cylindrical value were excluded.

## Statistical Analysis

Statistical analysis of the study’s data was conducted using SPSS version 21.0 (SPSS, Inc., Chicago, IL) and the Minitab 19.2 package programs. The normal distribution of continuous variables was assessed via the Kolmogorov‒Smirnov and Shapiro‒Wilk tests. On the basis of their distribution, variables are presented as either the mean ± standard deviation or median (interquartile range (IQR)). For comparisons between two dependent groups with normally distributed continuous variables, the paired t test was employed. In cases where a normal distribution was not observed, the Wilcoxon signed-rank test was utilized. A significance level of *p* < 0.05 was deemed statistically significant in all analyses.

## Results

A total of 112 eyes of 56 individuals, including 26 males with a mean age of 38.88 ± 7.00 years and 30 females with a mean age of 40.94 ± 7.76 years, were evaluated (*p* = 0.310). Six months following MBS, the average BMI in men decreased from 45.5 kg/m^2^ to 34 kg/m^2^ (*p* < 0.001), and the BMI decreased from 47 kg/m^2^ to 33 kg/m^2^ (*p* < 0.001) in women. The percent total weight loss (%TWL) was 27.19 ± 5.73 in men and 22.63 ± 4.31 in women (*p* < 0.001). The percentage of excess weight loss (%EWL) was 48.11 in men and 38.03 in women (*p* < 0.001). In both sexes, preoperative systolic and diastolic blood pressures, serum fasting glucose values, HbA1c levels, triglyceride levels, HDL cholesterol levels, and total cholesterol levels decreased significantly six months after MBS (for all, *p* < 0.001) (Table 1).

**Table 1:** Participant’s clinical characteristics before and after metabolic and bariatric surgery

	Male (*n* = 26)			Female (*n* = 30)		
	Before surgery	Six months after	*p* value	Before surgery	Six months after	*p* value
Weight (kg)	135.00 ± 20.10	94.88 ± 15.05	< 0.001	127.66 ± 15.82	89.16 ± 10.26	< 0.001
BMI (kg/m^2^), median (IQR)	45.50 (15)	34.00 (11)	< 0.001	47.00 (7)	33.00 (6)	< 0.001
Systolic BP (mmHg), median (IQR)	135.00 (8)	129.00 (10)	< 0.001	135.50 (12)	129.00 (12)	< 0.001
Diastolic BP (mmHg), median (IQR)	76.00 (10)	74.50 (6)	< 0.001	75.50 (7)	74.00 (6)	< 0.001
Glucose (mg/dL)	95.62 ± 14.98	87.96 ± 12.05	< 0.001	99.78 ± 11.87	91.53 ± 8.82	< 0.001
HbA1c (%, mmol/mol), median (IQR)	6.10 (0)	5.40 (1)	< 0.001	6.00 (0)	5.40 (0)	< 0.001
Triglyceride (mg/dL), median (IQR)	127.00 (16.75)	118.00 (24.75)	< 0.001	122.00 (16.50)	115.00 (17.25)	< 0.001
HDL (mg/dL), median (IQR)	41.00 (12.25)	41.50 (14.25)	0.001	42.00 (19.25)	44.00 (16.50)	0.001
LDL (mg/dL), median (IQR)	126.50 (42.25)	127.50 (38.75)	0.299	117.00 (46.75)	115.00 (45.25)	0.011
Total cholesterol (mg/dL)	156.33 ± 18.30	158.67 ± 18.11	0.002	145.88 ± 18.84	147.94 ± 18.20	< 0.001

*BMI* Body mass index; *BP* Blood pressure; *HbA1c* Glycosylated haemoglobin; *HDL* High-density lipoprotein; *LDL* Low-density lipoprotein; *IQR* İnterquartile range; Data are the means ± SDs

The mean ± standard deviation was used for normally distributed continuous variables, and the median (interquartile range) was used for nonnormally distributed continuous variables

Bolded *p* values are statistically significant

The average intraocular pressure before MBS was 18.25 mmHg in men and 18 mmHg in women. Six months after surgery, it decreased to 15.75 mmHg in men (*p* < 0.001) and 16.25 mmHg in women (*p* < 0.001). There was no significant difference in refractive error or best-corrected visual acuity between men and women when preoperative values were compared with those at the six-month postoperative follow-up in both eyes. Significant differences were detected between the average values of intraocular pressure, central corneal thickness, anterior chamber depth, volume and angle at the preoperative examination and the sixth-month postoperative follow-up in both genders (*p* < 0.001 for all) (Table 2).

**Table 2:** Changes in anterior segment parameters six months after metabolic and bariatric surgery

	Male (*n* = 26)			Female (*n* = 30)		
	Before surgery	Six months after	*p* value	Before surgery	Six months after	*p* value
İntraocular pressure (mmHg)	18.25 (6.38)	15.75 (4.00)	< 0.001	18.00 (4.63)	16.25 (3.00)	< 0.001
Central corneal thickness (μm)	536.75 (31.00)	527.00 (37.63)	< 0.001	541.69 ± 13.26	530.98 ± 13.04	< 0.001
Anterior chamber depth (mm)	2.43 (0.42)	2.85 (0.22)	< 0.001	2.39 (0.24)	2.83 (0.16)	< 0.001
Anterior chamber volume (mm^3^)	135.00 (11.00)	150.75 (11.88)	< 0.001	132.50 (6.88)	149.50 (10.25)	< 0.001
Anterior chamber angle (°)	36.93 (5.91)	37.53 (4.86)	< 0.001	36.53 (2.27)	37.33 (1.90)	< 0.001
Refractive error (right eye)	−0.25 (1.19)	−0.25 (1.25)	0.317	−0.25 (1.69)	−0.25 (1.50)	0.134
Refractive error (left eye)	0.00 (1.00)	0.00 (1.00)	1.00	−0.25 (1.25)	−0.13 (1.19)	0.725
Best-corrected visual acuity (Snellen equivalent) (right eye)	9.00 (2)	9.00 (2)	0.480	9.00 (2)	9.00 (2)	0.430
Best-corrected visual acuity (Snellen equivalent) (left eye)	1.00 (1)	9.00 (1)	0.366	9.00 (2)	9.00 (2)	0.557

*μm* micrometre; *mm* millimetre; ° degrees. Data are the mean ± SD

Bolded *p* values are statistically significant

**Fig. 1:**
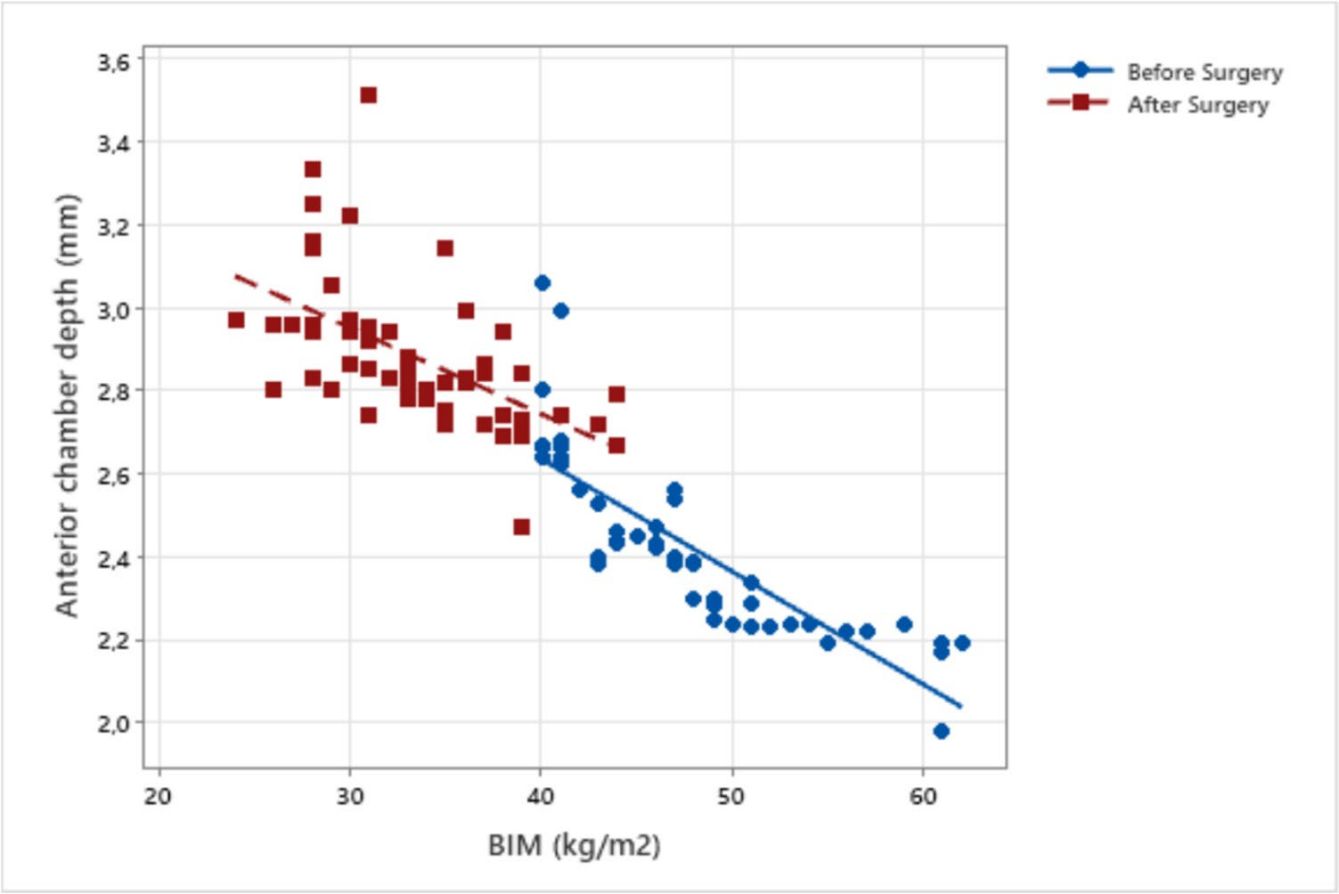
Correlation of BMI with anterior chamber depth before and after metabolic and bariatric surgery

**Fig. 2:**
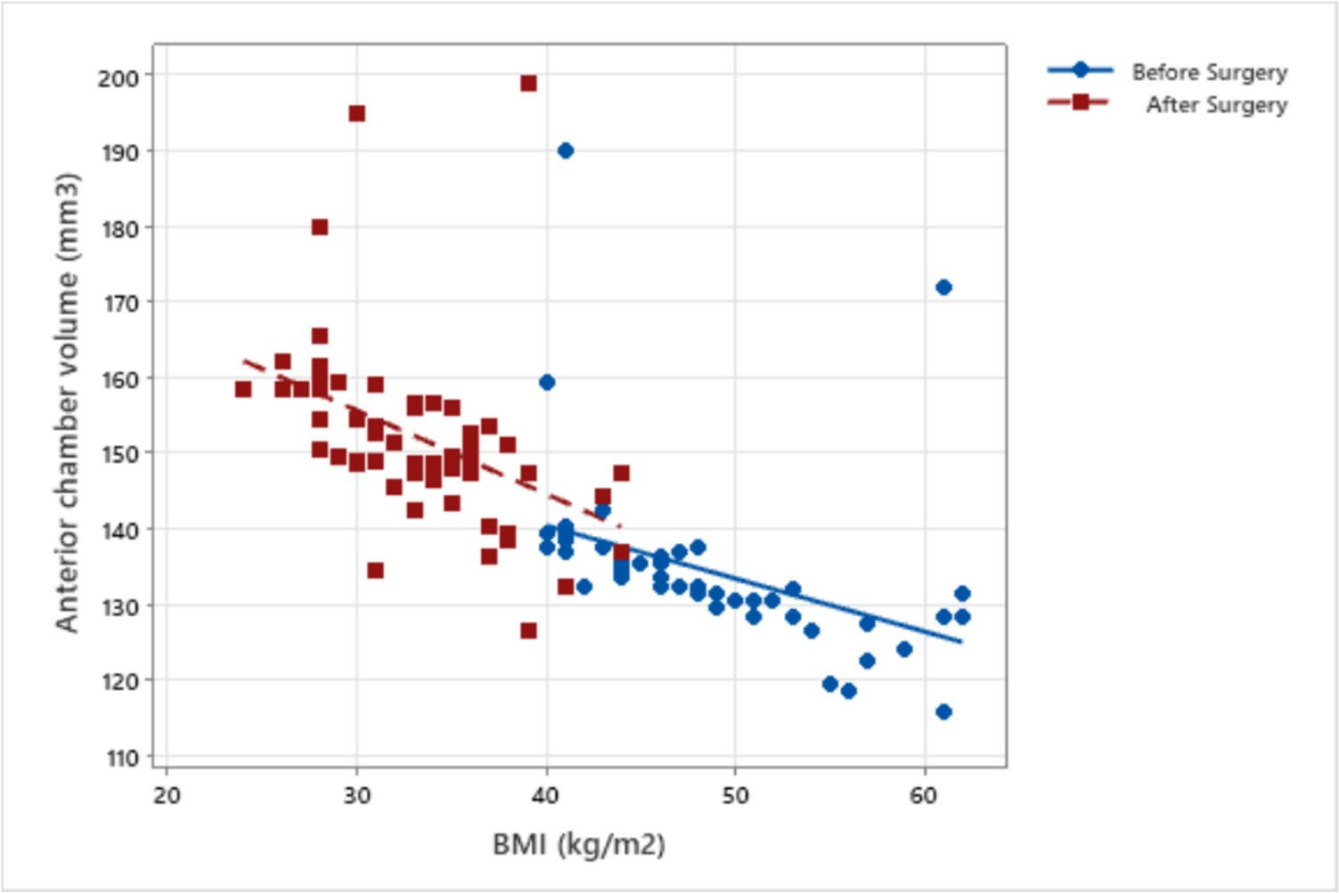
Correlation of BMI with anterior chamber volume before and after metabolic and bariatric surgery

**Fig. 3:**
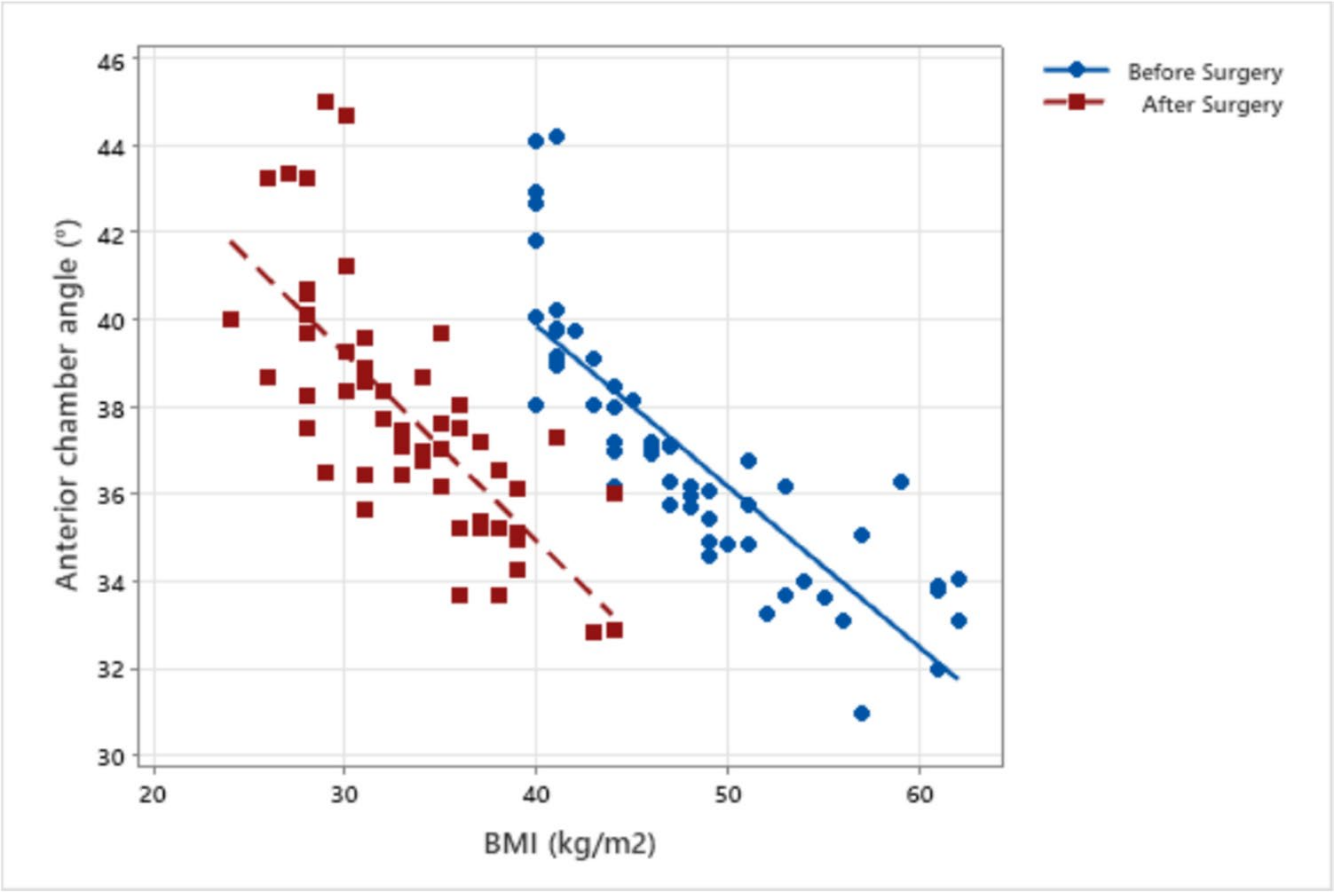
Correlation of BMI with anterior chamber angle before and after metabolic and bariatric surgery

According to the Spearman correlation test, a positive correlation was detected between BMI and both central corneal thickness and intraocular pressure, and a negative correlation was detected with anterior chamber depth, volume and angle in both genders before and after MBS (for all, *p* < 0.001) (Figs. 1, 2, and 3) (Table 3).

**Table 3:** Correlation coefficients of BMI with clinical parameters before and after metabolic and bariatric surgery

	Male (*n* = 26)		Female (*n* = 30)	
	Before surgery	Six months after	Before surgery	Six months after
İntraocular pressure (mmHg)	0.919 (< 0.001)	0.893 (< 0.001)	0.941 (< 0.001)	0.615 (< 0.001)
Central corneal thickness (μm)	0.913 (< 0.001)	0.913 (< 0.001)	0.834 (< 0.001)	0.784 (< 0.001)
Anterior chamber depth (mm)	−0.963 (< 0.001)	−0.807 (< 0.001)	−0.905 (< 0.001)	−0.540 (< 0.001)
Anterior chamber volume (mm^3^)	−0.721 (< 0.001)	−0.686 (< 0.001)	−0.889 (< 0.001)	−0.635 (< 0.001)
Anterior chamber angle (°)	−0.898 (< 0.001)	−0.915 (< 0.001)	−0.846 (< 0.001)	−0.591 (< 0.001)

*μm* micrometre; *mm* millimetre; ° degree

The numbers in parentheses are *p* values

Bolded *p* values are statistically significant

## Discussion

This study examined the impact of MBS on anterior segment parameters in obese individuals. The findings revealed a reduction in intraocular pressure and central corneal thickness, along with an increase in anterior chamber depth, volume and angle six months postsurgery. Additionally, a significant correlation was identified between changes in the anterior segment and the degree of BMI change. Notably, this study is the first to investigate the influence of MBS on anterior segment parameters and their interaction with BMI variations.

Research has consistently demonstrated that individuals with obesity exhibit elevated intraocular pressure compared with those within the normal range [1, 2, 5–9]. Numerous studies have confirmed a significant positive correlation between BMI and intraocular pressure [5, 7, 10, 11]. However, it is important to note that Trape et al. reported no significant difference in their study [12]. However, the specific pathophysiological mechanism underlying increased intraocular pressure in individuals with obesity remains unclear. Nevertheless, mechanical and cellular factors are believed to contribute to this phenomenon [1, 5, 13].

In the context of obesity, an observed increase in retrobulbar fat volume corresponds with increasing BMI. Stojanov et al. reported a positive correlation between increased retrobulbar fat volume and elevated intraocular pressure [8]. Moreover, increased adipose tissue has been reported to impact episcleral venous pressure, thereby influencing aqueous outflow. With obesity, there is an increase in blood viscosity and cardiovascular pathologies due to hyperlipidaemia and hyperglycaemia. These increases affect venous flow, causing the aqueous humour to encounter venous resistance, as it leaves the eye and makes its evacuation difficult [14]. Additionally, it has been proposed that heightened oxidative stress, increased leptin levels, and decreased ghrelin levels in individuals with obesity contribute to amplified oxidative DNA damage within the trabecular meshwork. This cascade leads to proteasome malfunction, induces trabecular meshwork degeneration, and ultimately increases outflow resistance [2, 10, 15].

Numerous studies evaluating the impact of MBS on intraocular pressure have reported a reduction in IOP. However, the exact underlying pathology remains unclear. Lam et al. emphasized that for every 10% reduction in weight achieved through MBS, there was a corresponding 1.4 mmHg decrease in intraocular pressure, highlighting the significance of orbital fat reduction [7]. Çekiç et al. reported an increase in retrobulbar and orbital blood flow following MBS, which coincided with a decrease in intraocular pressure [11]. Conversely, Zvia et al. reported a decrease in intraocular pressure after LSG surgery but did not establish a discernible correlation between the extent of weight loss and the reduction in intraocular pressure [16]. In this study, similar to the findings of Çekiç et al., we documented a significant reduction intraocular pressure among patients who underwent LSG [11]. Additionally, consistent with their investigation, we identified a positive correlation between the magnitude of weight loss and the decrease in intraocular pressure.

The existing body of research examining the connections among obesity, intraocular pressure, and anterior segment parameters is predominantly based on cross-sectional data. Studies demonstrating a tangible reduction in anterior segment parameters over time associated with obesity are limited. Similarly, few investigations have investigated the impact of substantial weight loss on both intraocular pressure and anterior segment parameters in individuals with obesity. Nevertheless, it is postulated that the increased volume of retrobulbar adipose tissue in obese individuals may lead to a reduction in anterior segment values, potentially due to compression between the bone and the orbit. For example, Guneş et al. reported that the anterior chamber depth was notably lower in individuals with obesity than in control individuals, with no significant disparities noted in anterior chamber volume or angle [9]. Conversely, Pannon et al. reported no significant distinctions in anterior chamber depth or angle between a control group and obese individuals [5].

Some studies have also indicated a tendency towards more hypermetropic refraction and shorter axial length in individuals with obesity [17, 18]. Moreover, numerous studies have reported an increased prevalence of cataracts in individuals with obesity. Anterior segment values, including anterior chamber depth, volume and angle, tend to decrease with the onset of cataracts and the thickening of the lens [2, 6]. However, the extent to which lens thickness may be altered in individuals with obesity and, as a result, MBS remains an area that requires further investigation. For this reason, patients with cataracts were excluded from our study. Tip 2 diabetes, hypertension and hyperlipidaemia are known risk factors for intraocular pressure. Some studies have shown that increases in the duration and severity of these metabolic diseases and increases in HbA1c and hyperlipemia affect corneal morphology and the anterior segment [1, 2]. In our study, patients with these disorders were excluded. However, in our study, significant decreases in blood sugar levels, arterial hypertension and lipid levels were observed in the postoperative period after MBS. It is hypothesized that the decrease in these values may influence intraocular pressure and the anterior segment.

Retroorbital fat tissue may change spontaneously with age and is thought to decrease with MBS. Consequently, owing to the decreased mechanical pressure exerted by the diminishing fat, we observed a reduction in intraocular pressure and an increase in anterior chamber depth, volume and angle in our study. Gulkaş et al. noted significant refractive changes in the eyes after one year of MBS, but anterior chamber depth did not significantly change [10]. In contrast, our study revealed significant changes in anterior chamber depth, volume and angle within six months. We attribute these differences to the utilization of distinct anterior segment imaging methodologies and variations in the BMI of the patient populations. Circulating cytokine or metabolic stress marker levels change with MBS. Changes in the cornea may occur due to changes in oxidative stress factors [19]. The levels of circulating cytokines or metabolic stress markers were not measured in our study. This was one of the limitations of our study. However, it is assumed that the levels of circulating cytokines or metabolic stress markers may have changed, and this may have a role in the change in anterior segment values by altering the corneal thickness and trabecular network. Regarding the correlation between BMI and anterior segment parameters, Guneş et al. reported a significant negative correlation between BMI and anterior chamber depth and angle, which aligns with our findings [9]. However, unlike our study, they did not find a significant correlation between BMI and anterior chamber volume.

Postbariatric surgery, patients often experience deficiencies in essential nutrients such as iron, selenium, zinc, and copper, as well as vitamins A, E, and B. Notably, vitamin A deficiency can lead to an increased incidence of pathologies such as corneal xerosis, keratomalacia, and corneal ulcers. The impact of malabsorption on central corneal thickness in individuals who have undergone MBS remains unclear [3]. Although various studies have demonstrated a significant correlation between BMI and intraocular pressure, the association between BMI and central corneal thickness is less definitive [5, 7, 10, 11]. Su et al. reported a positive correlation [20], but some studies have reported no significant correlation [1, 5, 9]. However, a correlation was noted between intraocular pressure and central corneal thickness, with greater central corneal thickness associated with greater intraocular pressure [5]. In this study, we observed substantial differences in central corneal thickness and intraocular pressure between the preoperative and postoperative groups. Similarly, Zvia et al. reported a significant decrease in both intraocular pressure and central corneal thickness following MBS [15]. Conversely, Gulkaş et al. reported no significant change in central corneal thickness after MBS [10]. These discrepancies in findings may stem from various factors, including differences in patient populations, surgical techniques, and follow-up durations.

### *Limitations of the Study*

The limitations of this study include its small sample size, the selection of participants from a single centre with a homogeneous population, and the relatively short follow-up period of six months. Furthermore, factors such as oxidative stress and metabolic parameters, which may influence anterior segment parameters, were not measured.

## Conclusion

In conclusion, this study revealed a decrease in intraocular pressure and central corneal thickness, along with an increase in anterior chamber depth, volume and angle following MBS. BMI was negatively correlated with anterior chamber depth, volume and angle. These findings underscore the necessity for vigilant postoperative management of intraocular pressure, precise refractive error assessment, and accurate intraocular lens calculations in patients who undergo cataract surgery subsequent to MBS. Bariatric surgeons and ophthalmologists should work with multidisciplinary teams for the treatment of such patients. To comprehensively understand the effects of MBS on anterior segment parameters, further studies are needed to examine long-term effects at the cellular level with new technological imaging devices.

## Data Availability

All the data generated or analysed during this study are included in this article. Further inquiries can be directed to the corresponding author.
